# Raffinose Family Oligosaccharides Act As Galactose Stores in Seeds and Are Required for Rapid Germination of *Arabidopsis* in the Dark

**DOI:** 10.3389/fpls.2016.01115

**Published:** 2016-07-26

**Authors:** Roman Gangl, Raimund Tenhaken

**Affiliations:** Department of Cell Biology, Division of Plant Physiology, University of SalzburgSalzburg, Austria

**Keywords:** *Arabidopsis thaliana*, galactose, galactinol, galactosylhydrolase, galactosyltransferases, raffinose, raffinose family oligosaccharides, raffinose synthase

## Abstract

Raffinose synthase 5 (AtRS5, At5g40390) was characterized from *Arabidopsis* as a recombinant enzyme. It has a far higher affinity for the substrates galactinol and sucrose than any other raffinose synthase previously reported. In addition raffinose synthase 5 is also working as a galactosylhydrolase, degrading galactinol, and raffinose under certain conditions. Together with raffinose synthase 4, which is predominantly a stachyose synthase, both enzymes contribute to the raffinose family oligosaccharide (RFO) accumulation in seeds. A double knockout in raffinose synthase 4 and raffinose synthase 5 (*ΔAtRS4,5*) was generated, which is devoid of RFOs in seeds. Unstressed leaves of 4 week old *ΔAtRS4,5* plants showed drastically 23.8-fold increased concentrations of galactinol. Unexpectedly, raffinose appeared again in drought stressed *ΔAtRS4,5* plants, but not under other abiotic stress conditions. Drought stress leads to novel transcripts of raffinose synthase 6 suggesting that this isoform is a further stress inducible raffinose synthase in *Arabidopsis*. *ΔAtRS4,5* seeds showed a 5 days delayed germination phenotype in darkness and an elevated expression of the transcription factor phytochrome interacting factor 1 (*AtPIF1*) target gene *AtPIF6*, being a repressor of germination. This prolonged dormancy is not seen during germination in the light. Exogenous galactose partially promotes germination of *ΔAtRS4,5* seeds in the dark suggesting that RFOs act as a galactose store and repress *AtPIF6* transcripts.

## Introduction

Seeds of higher plants store sugars presumably as carbon reserves for metabolism, as storage molecules for germination and as structural components for growth. Raffinose family oligosaccharides (RFOs) together with sucrose (Suc) constitute the most significant fraction of water-soluble carbohydrates (WSCs; Frias et al., 1999) and occupy a special position in the storage, transport, and stress physiology of *Arabidopsis*. RFOs are derivatives of Suc to which one galactosyl unit, leading to the formation of raffinose (Raf, Suc-[Gal]_1_), or respectively, two galactosyl units, leading to the formation of stachyose (Sta, Suc-[Gal]_2_) are added. RFOs with up to 13 galactosyl residues are known from some plant species. During exposure of *Arabidopsis* to abiotic stress, Raf is the only RFO to accumulate (Taji et al., 2002; Nishizawa et al., 2008; ElSayed et al., 2014), whereas Raf as well as Sta accumulate during seed development (Ooms et al., 1993; Bentsink et al., 2000; Nishizawa-Yokoi et al., 2008).

Galactinol synthase (GolS, *AtGS1-10*; EC 2.4.1.123) (Pharr et al., 1981) initiates RFO biosynthesis by catalyzing UDP-galactose and myo-inositol (Ino) to galactinol (Gol) (Liu et al., 1995; Liu et al., 1998). RFO biosynthesis proceeds by stepwise transfer of galactosyl units (Sengupta et al., 2015) involving raffinose synthase (RafS, *AtRS5*, EC 2.4.1.82) (Lehle et al., 1970; Lehle and Tanner, 1973) and stachyose synthase (StaS, *AtRS4*, EC 2.4.1.67) (Tanner and Kandler, 1968; Lehle and Tanner, 1973; Holthaus and Schmitz, 1991; Hoch et al., 1999; Peterbauer and Richter, 2001).

The RafS gene family consists of 5 members in *Arabidopsis* (*AtRS1*, *AtRS2*, *AtRS4*, *AtRS5*, and *AtRS6*) whereas *AtRS3* is a pseudogene. The common name RafS suggests a common biochemical function (Knaupp et al., 2011), the biosynthesis of Raf. However, biochemical investigations with *AtRS* enzymes rather suggest numerous enzymatic activities for *AtRS* isoforms which cannot be predicted by bioinformatics, but needs further biochemical and genetic experiments to determine their functions *in vivo* and *in vitro*. For the isoform *AtRS2* Egert et al. (2013) showed a α-galactosidase activity but obtained no evidence for a RafS. By knocking out the gene for *AtRS5* Zuther et al. (2004) obtained mutant plants which lack the abiotic stress induced accumulation of Raf. Different abiotic stress factors including, cold, water-deficit, high salinity, heat shock, and methyl viologen-induced oxidative stress were tested by Egert et al. (2013). It was concluded that *AtRS5* is the only RafS in *Arabidopsis* being responsible for stress induced Raf formation. Unexpectedly, knockout plants in *AtRS5* still contain about 50% Raf in seeds, suggesting the existence of at least a second *AtRS* activity contributing to the RFOs in seeds. We recently characterized *AtRS4* as a StaS in *Arabidopsis*. This group of enzymes is characterized by a unique domain insertion in the enzyme, which allows sequence based identification of this enzymatic activity. StaS is a multifunctional RFO synthase in *Arabidopsis* (Gangl et al., 2015) which can also synthesize Raf under certain conditions. Furthermore, StaS has intrinsic galactosylhydrolase activity acting on RFOs as well as on Gol. It becomes therefore clear that the name RafS is often misleading if one is interested in the biochemical function of individual members of this gene family. Furthermore, studies sometimes make predictions based on the gene expression of *AtRS* genes and simply assume that this is RafS but the before mentioned data suggest that a clear differentiation between isoforms and their functions is very critical.

Seed germination is regulated by both exogenous and endogenous factors. In consideration of seed germination the dualism of light, not only as an energy source, but also as a key environmental signal (Oh et al., 2004), holds a key position as an integrator of both exogenous and endogenous factors. Light triggered photosynthesis produces sugars which continue the integrator function on metabolite level either as sources of reduced carbon (Sairanen et al., 2012) or as signaling molecules (Rolland et al., 2006; Wind et al., 2010).

In *Arabidopsis* seeds, germination will occur if a germination-promoting wavelength of light is perceived (Penfield et al., 2010). In response to light, phytochromes - very important plant light sensors and detectors - are translocated to the nucleus and activate various transcription cascades, like a set of basic helix-loop-helix (bHLH) transcription factors also known as phytochrome interacting factors (PIFs) (Ni et al., 1998; Duek and Fankhauser, 2005), that lead to the regulation of various physiological processes such as seed germination (Chory et al., 1996; Neff et al., 2000; Sullivan and Deng, 2003). *AtPIF6*, which is expressed during seed development, was shown by Penfield et al. (2010) to be important in establishing the level of primary seed dormancy.

Here, we report on the cloning of *AtRS5* from *A. thaliana* and the biochemical characterization of the functional purified recombinant enzyme heterologously expressed in *E. coli* (AtRS, At5g40390) as a RafS, Raf and Gol specific galactosylhydrolase. We also isolated T-DNA insertion lines in *AtRS4* and *AtRS5*. A double knockout was obtained by crossing both single insertion lines. It lacks all RFOs in seeds. One of the phenotypes of *ΔAtRS4,5* seeds shows a WT-like germination in the light, but a strongly delayed germination in the dark, suggesting that RFOs act as a source of galactose (Gal) during germination, which is controlled by the *AtPIF6* transcription factor.

## Materials and Methods

### Plant Material and Growth Conditions

*Arabidopsis thaliana*, ecotype Columbia (wild type, WT), *ΔAtRS4*, *ΔAtRS5*, and *ΔAtRS4,5* seeds, were cold-treated and either grown in standard fertilized soil (type ED73), on 0.5% plant water agar plates (Duchefa), on 0.8% water agarose plates (LE-agarose; Biozym), on 0.3% water phytagel plates or on 0.5× MS plates [solid Murashige Skoog medium with 0.5× MS-salts (Duchefa), 0.3% (w/v) phytagel, KOH (pH 5.7)]. Plants grew in a short-day growth chamber with 60% humidity under standard control conditions (SCC) at 23°C with 8 h light (approximately 100 μE m^-2^ s^-1^) and at 18°C during the dark phase. To induce flowering, plants were transferred to a long-day growth chamber with 16 h light. Seeds were surface sterilized for dispersion on plates. Triplicates of 30 to 50 seeds were plated and imbibed for 10 min at SCC. Plates were incubated for 5 days in darkness or illuminated with either low intensity of red (660 nm) light for 5 days with 8 h light and 16 h dark period. In some experiments plates were illuminated with far-red (730 nm) light for 10 min and afterward incubated in darkness for 7 days. Germination was defined as the time between sowing and protrusion of the radicle. Hypocotyls were measured by using the Measurement tool of ImageJ software (Schneider et al., 2012). Samples were taken from seeds and leaves of *A. thaliana* WT, *ΔAtRS4*, *ΔAtRS5*, and *ΔAtRS4,5* plants. Three biological replicates were grown from each line and one sample was picked from each biological replicate. One seed sample consisted of 20 mg seeds and one leaf sample consisted of two medium-sized leaves of plants that were put into pre-weighted reaction tubes together with one 3 mm stainless steel ball. After harvesting the plant material, sample tubes were weighted and immediately stored in liquid nitrogen. The plant material was pulverized in liquid nitrogen-cooled Teflon carriers for 2 min and 30 Hz using a ball mill (Model PM200, Retsch).

### Plant Stress Treatment

Plants were grown under SCC as described above for 4 weeks. For cold stress (CS) treatment, plants were transferred into an acclimation chamber with identical settings to SCC but with a constant temperature of 4°C for a period of 14 days. For salinity stress (SS) treatment, plants were irrigated with 150 ml of a 100 mM NaCl solution every 2^nd^ day for up to 14 days. For drought stress (DS) treatment, water deficit was subsequently imposed by withdrawing water supply for 7 days under SCC. Strong plant stress treatments (Taji et al., 2002; Nishizawa et al., 2008; Egert et al., 2013) were abrupt imposed with longer time periods of stress treatment or higher salt concentration to avoid stress acclimation and ensure Raf accumulation, which is known to accumulate to high levels over the course of several days of salt, cold or drought exposure (Krasensky and Jonak, 2012).

### *ΔAtRS4*, *ΔAtRS5* and *ΔAtRS4,5* Genotyping

**FIGURE 1:**
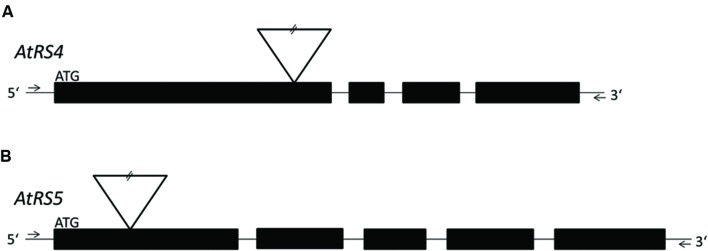
**Localization of T-DNA insertions.** For a better overview, already published data of T-DNA insertion localization in *ΔAtRS4* plants in Gangl et al. (2015) are shown in **(A)**. **(B)** shows the localization of T-DNA insertion site in *ΔAtRS5* plants. Exons are represented by black boxes and introns by lines. T-DNA is depicted by an inverted triangle.

*ΔAtRS4* plants carrying a T-DNA insert in the first exon of At4g01970 (**Figure 1A**; Gangl et al., 2015) in the Col-0 background were obtained from the SALK collection (At4g01970, [SALK_045237]) (Alonso et al., 2003). *ΔAtRS5* plants carrying a T-DNA insert in the first exon of At5g40390 (**Figure 1B**) in the Col-0 background were obtained from the GABI-Kat collection (At5g40390, [GABI-Kat_106F01]) (Kleinboelting et al., 2012). Genomic DNA (gDNA) was extracted from young *A. thaliana* leaves and genetic identity was determined by PCR technique. gDNA was extracted by the standard cetyl trimethylammonium bromide (CTAB) buffer method. Homozygous *ΔAtRS4*, *ΔAtRS5*, and *ΔAtRS4,5* plants were identified by PCR using primers listed in Supplementary Table S1.

### Extraction of WSCs

According to Lunn et al. (2006) liquid-liquid extraction was applied to extract WSCs and sugar alcohols from plant material. The pulverized plant material was incubated in quenching solution (chloroform to methanol in a 3:7 (v/v) ratio) at -20°C for 2 h. WSCs were extracted twice by adding 400 μl water and collected after centrifugation. The aqueous phases were vaporized using a vacuum concentrator (Model 5301 Concentrator plus, Eppendorf). After vaporization, all samples of each biological replicate were reconstituted and diluted in water.

### Cloning of *AtRS5*

Total RNA was extracted from *A. thaliana* leaves of by TriReagent buffer method according to Chomczynski (1993). Residual DNA was removed by treatment with RNase-free DNase (Thermo Scientific). Single strand cDNA was synthesized from 1 μg of total leaf RNA using the RevertAid First Strand cDNA synthesis kit (Thermo Scientific). For cloning the *AtRS5* gene based on the cDNA sequence of *A. thaliana* two primers with restriction sites were designed, AtRS5_fwd with Kpn I and AtRS5_rev with Sma I restriction site (Supplementary Table S1). PCR was performed with Phusion High-Fidelity DNA polymerase (Thermo Scientific) under following conditions: initial denaturation at 98°C for 30 s, followed by 5× (98°C for 10 s, 58°C for 30 s, 72°C for 90 s), 14× (98°C for 10s, 58+1°C for 30 s, 72°C for 90 s), 16× (98°C for 10 s, 72°C for 30 s, 72°C for 90 s) and final elongation at 72°C for 10 min. The PCR product was restricted with Kpn I and Sma I (Fast Digest; Thermo Scientific), gel purified using GeneJet Gel Extraction Kit (Thermo Scientific) and ligated into *E. coli* expression vector pQE30 (Qiagen) using Rapid DNA Ligation Kit (Thermo Scientific).

### Heterologous Expression and Functional Purification of Recombinant AtRS5

For heterologous expression of recombinant AtRS5 in *E. coli*, cells were cultivated at 37°C in a 1 L Erlenmeyer flask containing 75 ml LB medium supplemented with 100 μg ml^-1^ of ampicillin to an OD_600_ of 0.8 under vigorous shaking. The culture was cooled to 18°C and expression induced by addition of 1 mM isopropyl-β-D-thiogalactopyranoside (IPTG). After 20 h vigorous shaking at 18°C, the culture was cooled to 4°C for 30 min. The culture was split into three parts of 25 ml each, harvested and pellet was stored on -80°C. For functional purification of recombinant AtRS5, the pellet was resuspended in 2 ml of equilibration buffer (50 mM NaH_2_PO_4_, 300 mM NaCl, 1 mM DTT and a pH of 8.0 adjusted with NaOH). Lysozyme from chicken egg (final concentration of 1 mg ml^-1^) was added (Roche Applied Science) and gently shaken for 30 min on ice. After incubation, the cell suspension was sonicated with 60% amplitude (10 × 10 s bursts with 10 s cooling between each burst) on ice (Bandelin). For functional purification of His-tagged recombinant AtRS5, the clarified lysate was applied to pre-packed Protino^®^ Ni-TED 1000 columns (Macherey and Nagel) equilibrated with 2 ml equilibration buffer. The column was washed three times with 2 ml of equilibration buffer and bound recombinant protein was eluted with 1.5 ml of elution buffer (50 mM NaH_2_PO_4_, 300 mM NaCl, 1 mM DTT, and 250 mM imidazole and a pH of 8.0). Recombinant AtRS5 was analyzed by SDS-PAGE and immediately used in enzyme assays.

### Protein Determination and SDS-PAGE

Recombinant protein concentration was determined using the of Bradford assay method with bovine serum albumin as reference on a NanoDrop^®^ ND-1000 Spectrophotometer (peqLab). Different fractions, obtained during purification of recombinant AtRS5, were separated on a 10% SDS-PAGE gel and analyzed after colloidal Coomassie blue staining.

### RafS Enzyme Activity Assay

For RafS enzyme activity products were detected on a specialized HPLC system (high performance anion exchange chromatography with pulsed amperometric dection; HPAEC-PAD). RafS enzyme assays were carried out in 0.2 ml reaction tubes at a final volume of 100 μl containing 25 mM KH_2_PO_4_ (pH 7), 600 μM Suc, 100 μM Gol, and 650 ng of recombinant AtRS5. RafS enzyme assays were incubated in a PCR cycler (Peqlab) at 25°C for 60 min and reactions were stopped by heating the tubes to 95°C for 5 min. For RafS enzyme activity analysis, 10 μl of RafS enzyme assays were injected on the HPLC. In RafS enzyme assay, recombinant AtRS5 was assumed to catalyze the reaction Suc + Gol → Raf + Ino. Determination of RafS enzyme activity and biochemical data like buffer system optimum, effect of different pH values on RafS enzyme activity, temperature optimum, and enzyme kinetics of the recombinant AtRS5 were performed by measurements of Raf product formation.

### Gol, Raf, and Sta Galactosylhydrolase Enzyme Activity Assay

Detection of galactosylhydrolase enzyme activity was performed by product analysis on HPLC. Galactosylhydrolase enzyme assays were carried out in 0.2 ml reaction tubes at a final volume of 100 μl containing 25 mM KH_2_PO_4_ (pH 7), 650 ng of recombinant AtRS5, and 1 mM of different substrates (either Gol, Raf or Sta). Galactosylhydrolase enzyme assays were incubated in a PCR cycler at 25°C for 60 min and reactions were stopped by heating the tubes to 95°C for 5 min. For galactosylhydrolase enzyme analysis, 10 μl of galactosylhydrolase enzyme assays were injected on HPLC. In galactosylhydrolase enzyme assay, recombinant AtRS5 was assumed to catalyze the reaction Gol → Gal + Ino, reaction Raf → Suc + Gal, and reaction Sta → Raf + Gal. Determination of galactosylhydrolase activity and of enzyme kinetics of the recombinant AtRS5 were performed by measurements of Gal product formation.

### Detection of WSCs on HPLC System

Separation of WSCs produced during enzyme assays as well as WSCs extracted from plant material was performed on a ICS3000 system (Dionex Corporation), consisting of an ICS3000 single pump, ICS3000 electrochemical detector and a Dionex AS50 autosampler. A Dionex Carbopac PA20 column (150 × 3 mm i.d., 6.5 μm particle size) with a PA20 guard column (30 × 3 mm i.d., 6.5 μm particle size) was used for baseline separation of Gal, Suc, Raf, Sta, and Verb as well as separation of Ino and Gol. To achieve a baseline separation of Ino and Gol, a Dionex Carbopac MA1 column (250 × 4 mm i.d., 7.5 μm particle size) with a MA1 guard column (50 × 4 mm i.d., 7.5 μm particle size) was used. Sample measurements were performed as described in Gangl et al. (2015). During measurements the column oven was set to 30°C. The mobile phase consisted of solvent A, 200 mM NaOH, and solvent B, 15 mM NaOH. For chromatography of WSCs, data were analyzed with Chromeleon 7.12 (Thermo Scientific).

### Semi-Quantitative PCR (sqPCR)

sqPCR was carried out in 30 μl reactions with 1U recombinant Taq polymerase, at a primer annealing temperature of 55°C for 35 cycles. sqPCR primer (Supplementary Table S1) were designed using QuantPrime (Arvidsson et al., 2008). Target sequence was used to amplify a fragment of the corresponding cDNA. sqPCR product sizes were checked on 2% agarose gel and simultaneously performed no-template controls lead to no formation of sqPCR products. Three biological replicates were measured in triplicates.

### Quantitative PCR (qPCR)

Total RNA was extracted from *A. thaliana* seeds and leaves with the Agilent RNA Isolation Mini Kit (Agilent Technologies). cDNA was created as described above. qPCR triplets and no template control were performed on a AriaMx Real-Time PCR System (Agilent Technologies) with PCR product detection based on a SYBR Green fluorescence method. The qPCR program cycles through 10 s at 95°C, 30 s at 55°C, and 15 s at 72°C for 40 times. qPCR primer (Supplementary Table S1) were designed using QuantPrime (Arvidsson et al., 2008). *AtEF1α* was used as housekeeping gene for normalization and internal control. Agilent AriaMx software was used to analyze qPCR data using the relative quantification 2^-(ΔΔ^ ^Ct)^-method to evaluate quantitative variations. Three biological replicates were measured in triplicates.

## Results

### Sequence Analysis

Since we were searching for the second seed-specific RafS next to *AtRS5* as suggested by Egert et al. (2013), we performed a sequence alignment (Carmi et al., 2003) with Clustal Omega (Sievers et al., 2011) of all five *AtRS* amino acid sequences encoded by genes annotated in the *Arabidopsis* genome (Supplementary Figure S1). The phylogenetic tree (Supplementary Figure S2B) placed the RFO synthases [*AtRS4* (AtSTS, At4g01970) and *AtRS5* (AtSIP1-like, At5g40390)] in a cluster separated from the alkaline α-galactosidases/SIPs [*AtRS1* (AtSIP1, At1g55740) and *AtRS2* (AtSIP2, At3g57520)]. Based on this phylogenetic separation and published biochemical data (Peters et al., 2010; Egert et al., 2013; Gangl et al., 2015), we previously hypothesized *AtRS4* as the second seed-specific RafS (Gangl et al., 2015). Furthermore, the phylogenetic tree placed *AtRS6* (AtDIN10, At5g20250) into neither the group of alkaline α-galactosidase/SIP nor the group of RFO synthase despite very high sequence identity and similarity (Supplementary Figure S2A), which makes a classification of *AtRS6* very difficult solely based on bioinformatic data analysis.

### Cloning and Expression of *AtRS5*

**FIGURE 2:**
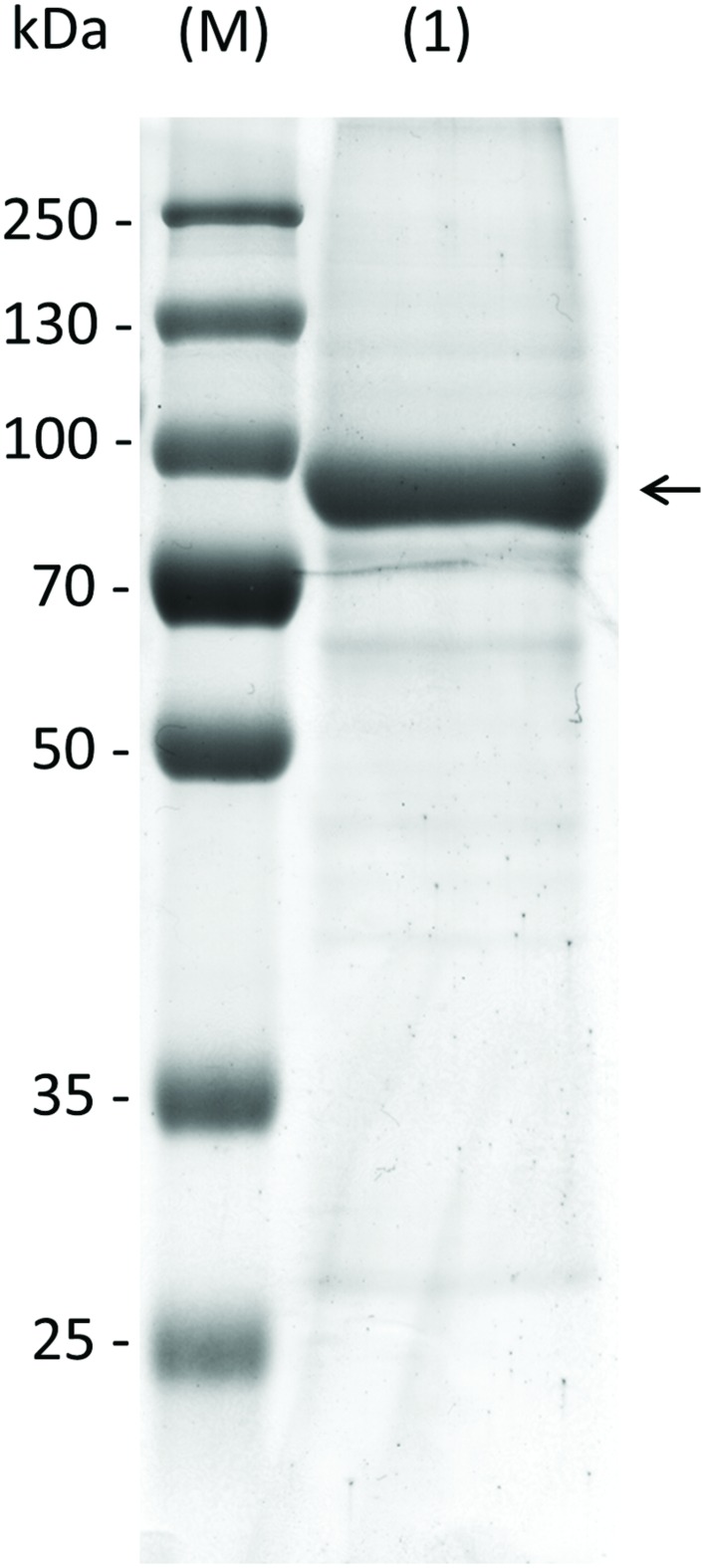
**Heterologous expression of recombinant AtRS5 in *E. coli*.** Eluate of the recombinant purified AtRS5 was analyzed on 10% SDS-PAGE. Lane (M), molecular weight marker; Lane (1), recombinant purified AtRS5.

Knockout plants in AtRS5 were previously shown to lack stress induced RFOs (Egert et al., 2013). However, the enzyme was never biochemically characterized and therefore the catalyzed reactions by AtRS5 are unclear. To confirm the biochemical function of *AtRS5* we cloned the full-length open reading frame of *AtRS5* cDNA, encoding 783 amino acids with a calculated molecular mass of about 86.24 kDa, into the *E. coli* expression vector pQE30 with a His-tag for convenient purification of recombinant AtRS5. We obtained soluble and functional protein. A single band of about 90 kDa on SDS-PAGE of the purified soluble recombinant protein is shown in **Figure 2**. Expression of recombinant AtRS5 in *E. coli* culture produced 43 mg of the recombinant protein per liter of cell culture with a specific RafS enzyme activity V_max_ (Suc) of 2.05 nkat mg^-1^ protein.

### Enzyme Assay

A RafS enzyme assay was optimized by testing a set of stabilizing co-factors (1 mM Mg^2+^, 1 mg ml^-1^ BSA, 0.1% Triton, 0.1% Tween 20, and 1 mM DTT). To stabilize the RafS enzyme activity during the purification procedure we added 1 mM DTT to the equilibration and elution buffer, which was sufficient to stabilize RafS enzyme activity without further need of DTT in the RafS enzyme assay. Recombinant AtRS5 showed a detectable RafS enzyme activity by Raf product formation when 650 ng of recombinant AtRS5 were incubated in 25 mM K-phosphate (KOH, pH 7) with 100 μM Gol and 600 μM Suc in 100 μl enzyme assays for 1 h at 25°C. Recombinant AtRS5 catalyzed under these conditions the conversion of Suc and Gol into Raf. The Raf product formation (Supplementary Figure S3) is dependent on the presence of Suc and Gol and identical with the commercially available reference compound. Concomitant, the product Ino accumulates linearly with Raf product formation. These experiments confirm that the recombinant AtRS5 is indeed a RafS.

### RafS Characterization

**FIGURE 3:**
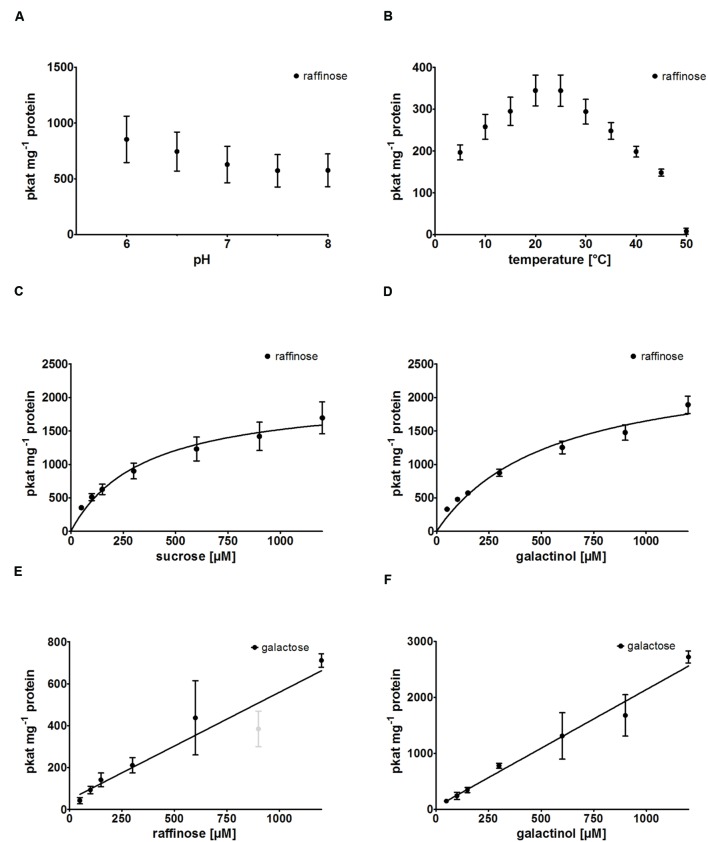
**Biochemical characterization of recombinant AtRS5. (A)** The dependence of RafS enzyme activity on different pH values and **(B)** the effect of reaction temperature on RafS enzyme activity were measured. Recombinant AtRS5 enzyme activity was measured with substrate concentrations from 50 to 1,200 μM under standard conditions for 60 min. **(C)** Substrate saturation by Michaelis-Menten curve for Suc is shown. **(D)** Substrate saturation by Michaelis-Menten curve for Gol is shown. **(E)** Substrate saturation for Raf hydrolysis measuring Gal is shown. **(F)** Substrate saturation curve for Gol hydrolysis measuring Gal is shown. Hydrolysis rates show a linear increase with substrate concentration. Raf and Gal concentrations were determined using enzyme assay. Values are averages from three independently performed assays (±SD). Excluded data point is indicated by gray color.

RafS enzyme activity of recombinant AtRS5 was determined in different buffer systems at pH 7 and at different pH values ranging from pH 6.0 to 8.0 (**Figure 3A**), using Raf product formation as the detection method. A slightly higher activity was obtained at pH 6 compared to pH 7, but the activity clearly drops under alkaline conditions. We therefore performed enzyme assays at a neutral pH of 7 to simulate cytosolic conditions, which has roughly 75% of the maximal RafS enzyme activity. Potassium phosphate puffer is superior to sodium phosphate buffer and MES-buffer. Recombinant AtRS5 showed RafS enzyme activity within a temperature range from 5 to 50°C (**Figure 3B**). The temperature optimum was located around 20 to 25°C. At 5°C still 57% of RafS enzyme activity was sustained, whereas 97.7% of the RafS enzyme activity was lost at 50°C caused by protein inactivation.

### Substrate Specificity and Product Formation

**FIGURE 4:**
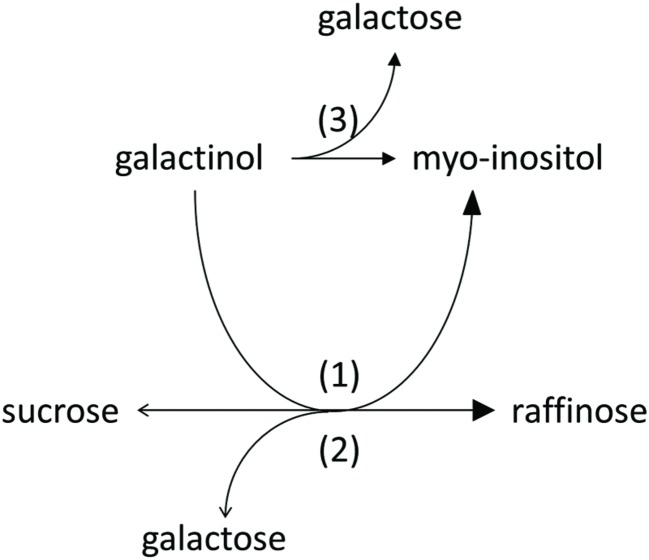
**Enzyme activities of recombinant AtRS5.** Recombinant AtRS5 annotated as putative RafS (AtRS, AtSIP-like, At5g40390) showed a high affinity RafS (1), as well as Raf (Suc-[Gal]_1_) (2), and Gol (3) specific galactosylhydrolase enzyme activity.

We observed product formation of Ino and Gal in enzyme assay controls offering recombinant AtRS5 only Gol, leading to the conclusion that recombinant AtRS5 possesses a galactosylhydrolase enzyme activity. Therefore, we tested the galactosylhydrolase enzyme activity offering recombinant AtRS5 either Sta, Raf or Gol. Galactosylhydrolase enzyme activity could only be observed offering recombinant AtRS5 Raf or Gol (**Figure 4**). The presented results indicate that *AtRS5* has a high affinity RafS (**Figures 3C,D**) as well as a Raf (**Figure 3E**) and Gol specific galactosylhydrolase enzyme activity (**Figure 3F**).

### Kinetic Analysis

Kinetic analysis of the RafS enzyme activity of recombinant AtRS5 was performed for the substrates Suc and Gol. The enzyme kinetic of recombinant AtRS5 for Suc (**Figure 3C**) showed a hyperbolic curve from which a *K*_m_ value for Suc of 348 ± 69 μM and a *V*_max_ value of 2,050 ± 154 pkat mg^-1^ protein was calculated using curve regression analysis with SigmaPlot 13.0 software. Substrate saturation curves of recombinant AtRS5 for Gol (**Figure 3D**) followed a hyperbolic curve according to Michaelis-Menten kinetic. The K_m_ value of recombinant AtRS5 for Gol in a substrate range from 50 to 1,200 μM was calculated as 548 ± 118 μM and a *V*_max_ value of 2,554 ± 247 pkat mg^-1^ protein.

Kinetic analysis of the galactosylhydrolase enzyme activity of recombinant AtRS5 was performed for the substrates Raf and Gol. AtRS5 has a hydrolase activity toward Raf (**Figure 3E**) and Gol (**Figure 3F**), which increase linear with substrate concentration.

### Knockout Plants of *ΔAtRS4, ΔAtRS5*, and *ΔAtRS4,5*

**FIGURE 5:**
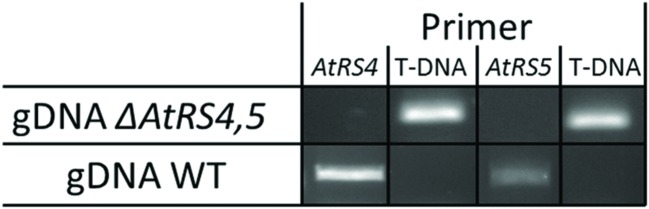
***ΔAtRS4,5* genotyping.** Insertion of T-DNA was determined by PCR product obtained from gDNA of WT and *ΔAtRS4,5* leaves.

Zuther et al. (2004) already identified homozygous *ΔAtRS5* plants from the GABI-Kat_106F01 line and performed Southern blotting experiments indicating only one T-DNA insertion in the *ΔAtRS5* genome. In *ΔAtRS5* plants transcript for *AtRS5* were absent in control as well as in stressed plants. In order to analyze the regulatory network of the RFO physiology in seeds of *A. thaliana*, we produced a *ΔAtRS4,5* double knockout from cross-fertilization of the two T-DNA-insertion lines SALK_045237 (*ΔAtRS4*) and GABI-Kat_106F01 (*ΔAtRS5*). The homozygous *ΔAtRS4* was crossed with a homozygous *ΔAtRS5*, selfed and homozygous *ΔAtRS4,5* double knockout was isolated and characterized by PCR for homozygous T-DNA insertion in *AtRS4* and *AtRS5* genes (**Figure 5**).

### Changes of WSC Extracts from *ΔAtRS4,5* Seeds and Leaves

**FIGURE 6:**
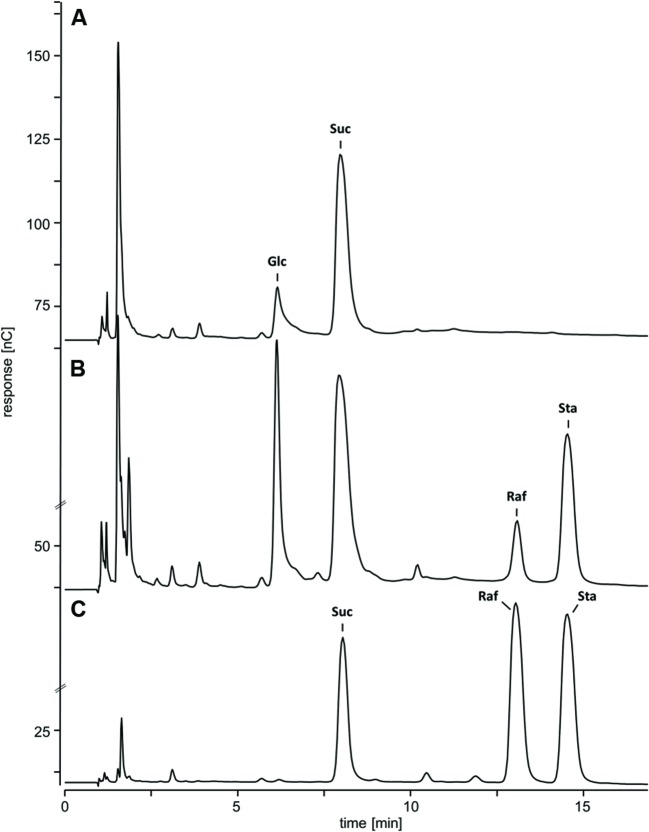
**Chromatogram of metabolic phenotype of *ΔAtRS4,5* seeds.** HPAEC-PAD chromatogram of WSCs and sugar alcohols extracted from **(A)** *ΔAtRS4,5* and **(B)** WT seeds, and **(C)** 100 μM Suc, Raf, and Sta as reference compounds.

**FIGURE 7:**
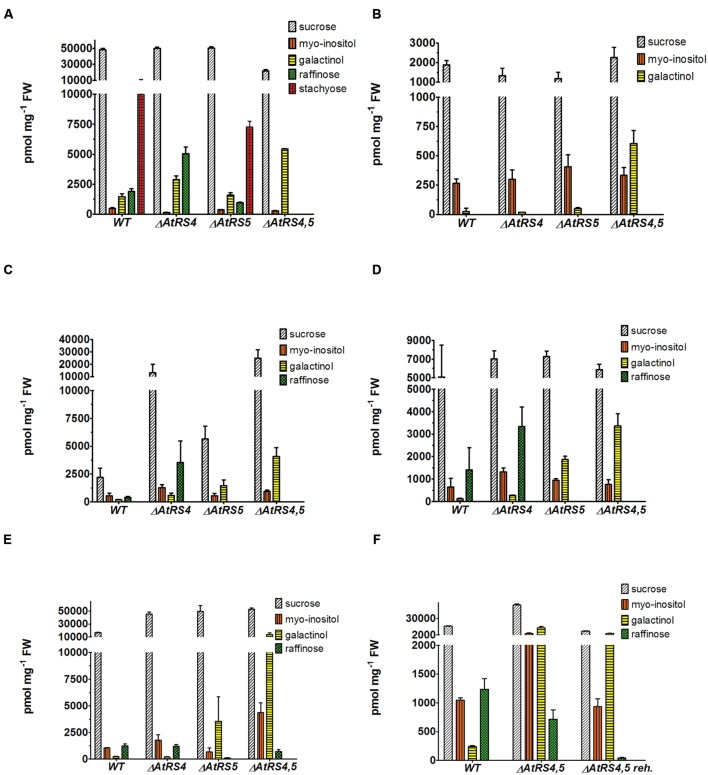
**Metabolites in WT*, ΔAtRS4*, *ΔAtRS5*, and *ΔAtRS4,5* plants.** Metabolites in **(A)** seeds, **(B)** leaves of SCC treated plants, **(C)** leaves of SS treated plants, **(D)** leaves of CS treated plants, **(E)** leaves of DS treated plants, and **(F)** leaves of rehydrated plants. For entire comparison of metabolites between mutants, already published data of metabolite measurements of WT and *ΔAtRS4* plants in **(A)** seeds and **(B)** leaves of SCC treated plants in Gangl et al. (2015) **Figure 10B** are shown. Values are averages of three independently performed measurements (±SD).

Water-soluble carbohydrates were extracted from WT (**Figure 6B**) and *ΔAtRS4,5* (**Figure 6A**) seeds, and analyzed by HPLC. *ΔAtRS4,5* seeds showed a total loss of detectable RFO metabolites, respectively, Raf and Sta (**Figure 7A**). In *ΔAtRS4,5* seeds, Gol concentration was increased 3.7-fold compared to WT seeds. Furthermore, *ΔAtRS4,5* seeds showed Suc concentration decreased by a factor of 0.4, and unexpectedly glucose (Glc) concentration decreased by a factor of 0.2 (**Figure 6A**).

Water-soluble carbohydrates were extracted from WT, *ΔAtRS4*, *ΔAtRS5*, and *ΔAtRS4,5* leaves and analyzed by HPLC (**Figure 7B**). Like with Suc concentrations, those of Ino were about equal in all genotypes. In *ΔAtRS4,5* leaves compared to WT leaves, Gol (604 ± 112 pmol mg^-1^ FW) concentration was 23.8-fold increased.

### WSC Extracts from DS Treated *ΔAtRS4,5* Leaves Contain Again Raf

Water-soluble carbohydrates were extracted from 4 week old control plants grown under SCC, with 100 mM NaCl for 14 days SS treated, at 4°C for 14 days CS treated and by withdrawing water for 7 days DS treated leaves of WT and *ΔAtRS4*, *ΔAtRS5*, and *ΔAtRS4,5* plants and analyzed by HPLC (**Figures 7C–E**). Leaves of SCC treated plants showed no Raf accumulation. As expected all stress treated leaves of WT (SS 411 ± 92 pmol mg^-1^ FW, CS 1,409 ± 987 pmol mg^-1^ FW, DS 1,235 ± 183 pmol mg^-1^ FW) and *ΔAtRS4* (SS 3,524 ± 202 pmol mg^-1^ FW, CS 3,333 ± 871 pmol mg^-1^ FW, DS 1,218 ± 135 pmol mg^-1^ FW) plants showed Raf accumulation, whereas leaves of *ΔAtRS5* (DS 115.3 ± 12.5 pmol mg^-1^ FW) plants showed almost no detectable Raf accumulation. As expected SS and CS treated leaves of *ΔAtRS4,5* plants lacked Raf accumulation following the hypothesis of one single abiotic stress induced *AtRS* isoform (Egert et al., 2013). But surprisingly we still could detect Raf accumulation (DS 714 ± 163 pmol mg^-1^ FW) in leaves of DS treated *ΔAtRS4,5* plants (**Figure 7E**). To confirm this result we repeated DS treatment and rehydrated *ΔAtRS4,5* plants for 5 days. Indeed, we could detect the Raf accumulation again and even monitor the degradation of Raf (DS 43.1 ± 6.8 pmol mg^-1^ FW) in rehydrated leaves of *ΔAtRS4,5* plants (**Figure 7F**).

### sqPCR, qPCR, and Expression Analysis

**FIGURE 8:**
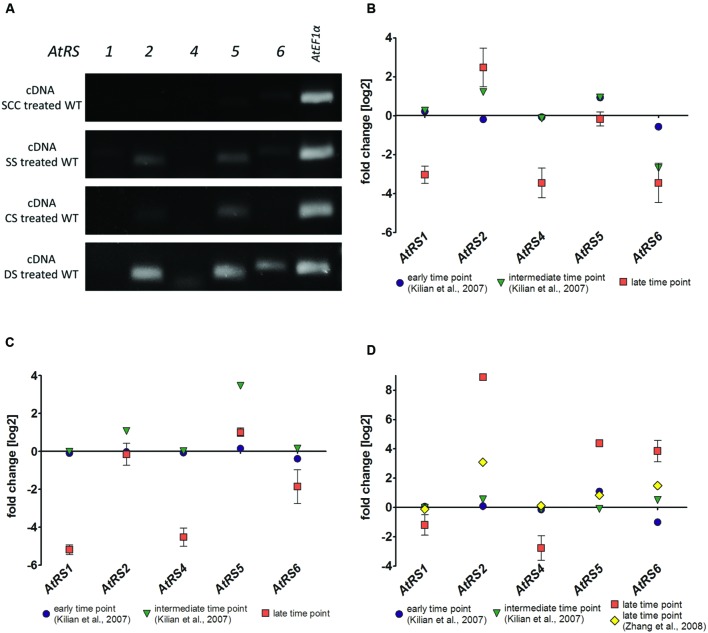
**sqPCR, qPCR and gene expression analysis of WT plants after different stress treatments. (A)** sqPCR of *AtRS1*, *AtRS2*, *AtRS4*, *AtRS5*, *AtRS6*, and *AtEF1α* was performed with reverse transcribed cDNA from WT leaves after SCC and different stress [including salt stress (SS), cold stress (CS) and drought stress (DS)] treatments. qPCR of *AtRS1*, *AtRS2*, *AtRS4*, *AtRS5*, *AtRS6*, *AtEF1α* was preformed and *AtRS* gene expressions were analyzed with Genevestigator (Hruz et al., 2008). The log2 fold change values are depicted after **(B)** salt, **(C)** cold, and **(D)** drought stress treatment. Gene expression data points are derived from microarray data from AtGenExpress (Schmid et al., 2005; Kilian et al., 2007) [early (up to 1 h) and intermediate (up to 24 h) time point from SS time course; early and intermediate time point from CS time course; early and intermediate time point from DS time course] and from Zhang et al. (2008) [late (up to 11 days) time points from DS] as verification. qPCR data points are generated from **(B)** 100 mM NaCl for 14 days (late time point) SS treated WT leaves, from **(C)** at 4°C for 14 days (late time point) CS treated leaves and from **(D)** DS treated leaves by withdrawing water for 7 (late time point) days.

The transcription of all five *AtRS* isoforms in WT leaves either grown untreated as control or after different stress treatments including SS, CS, and DS treatment were studied by using sqPCR. In control WT leaves, no sqPCR transcripts could be detected for all 5 *AtRS* isoforms. In SS, CS, and DS treated WT leaves, sqPCR showed a product for *AtRS5* transcript, as well as a specific PCR product for *AtRS2* transcript. Furthermore, sqPCR with DS treated WT leaves samples showed a PCR product specific for *AtRS6* transcript (**Figure 8A**). sqPCR product patterns of *AtRS* genes derived from different stress treatments were comparable to performed qPCR (late time point) and expression studies obtained from microarray data (**Figures 8B–D**) in Genevestigator (Hruz et al., 2008). Log2 fold change values above 1 lead to sqPCR product formation and confirmed our sqPCR results. Microarray data displayed *AtRS* gene expression levels dependent on different abiotic stress treatment and time period of exposure to abiotic stress treatments.

### *AtPIF6* Is Upregulated in *ΔAtRS4,5* Seeds

**FIGURE 9:**
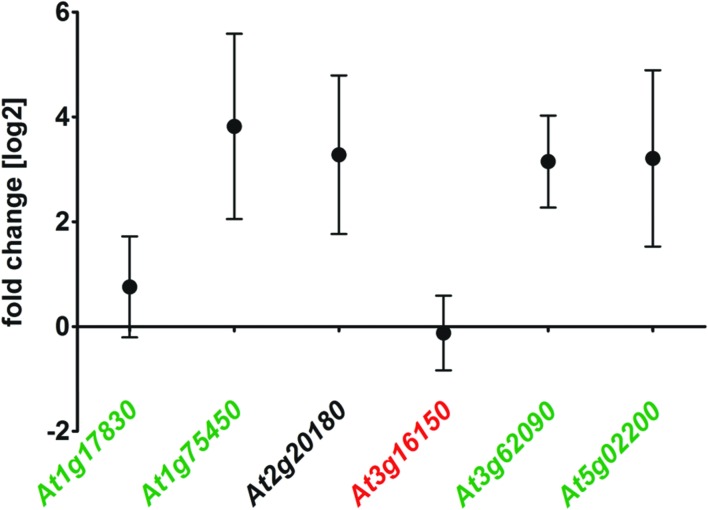
**Gene expression of *AtPIF1* target genes in *ΔAtRS4,5* compared to WT seeds.** At1g17830, At1g75450, At3g16150, *AtPIF6* (At3g62090), and *AtFHL* (At5g02200) are considered to be target genes of *AtPIF1* (At2g20180) (Oh et al., 2009). At1g17830, At1g75450, *AtPIF6* as well as *AtFHL* are upregulated target genes (green) of *AtPIF1* (black). At3g16150 is repressed in *AtPIF1* overexpressors (red), which is only slightly visible for At3g16150 in *ΔAtRS4,5*.

The delay of *ΔAtRS4,5* germination occurred only in darkness (Supplementary Figures S4 and S5). White light, as well as red light was able to rescue *ΔAtRS4,5* germination. After 5 days treatment with red light, 66.7% of WT seeds and 100% of *ΔAtRS4,5* seeds had germinated. Photo-reversible seed germination is controlled by phytochromes and eliminated in *ΔAtPIF1* seeds (Shinomura et al., 1994; Oh et al., 2004). Treatment with far-red light for 10 min continued by incubation in the dark fully blocks the germination of all genotypes for more than 7 days, suggesting that the phytochrome dependent steps in germination are working properly in all genotypes. Active phytochrome phosphorylates PIF-transcription factors causing ubiquitination and subsequent their degradation which results in the promotion of germination (Oh et al., 2004, 2009; Leivar and Quail, 2011). *AtRS* gene interaction analysis with ThaleMine from the Araport (Krishnakumar et al., 2015), revealed that *AtRS5* and *AtRS6* interact with *AtUBQ3* (polyubiquitin 3, At5g03240) (Kim et al., 2013), which is modulated by UV-B and light/dark treatments. Oh et al. (2009) identified direct target genes of *AtPIF1* (At2g20180). We therefore tested whether At1g17830, At1g75450, *AtPIF6* (At3g62090), and *AtFHL* (At5g02200), which are upregulated targets of *AtPIF1*, and/or At3g16150, which is a downregulated target of *AtPIF1*, are up, respectively, down regulated in *ΔAtRS4,5* seeds (**Figure 9**). qPCR experiments showed indeed a 7 to 8-fold increase in *AtPIF6* transcripts in *ΔAtRS4,5* seeds.

### Seed Germination Experiments

To test whether *ΔAtRS4,5* seeds show any differences in the germination kinetic, we put WT and *ΔAtRS4,5* seeds either on 0.5% water agar, or 0.8% water agarose plates to avoid dormancy breaking signals in plate media and monitored the germination rate under SCC at 23°C with 8 h light. *ΔAtRS4,5* seeds started to germinate after 2 days, while WT seeds showed a slightly delayed germination (Supplementary Figure S6). Faster *ΔAtRS4,5* germination kinetic resembled higher germination frequencies of *AtPIF6* overexpressor seeds between 15 and 25°C under light conditions (Penfield et al., 2010).

**FIGURE 10:**
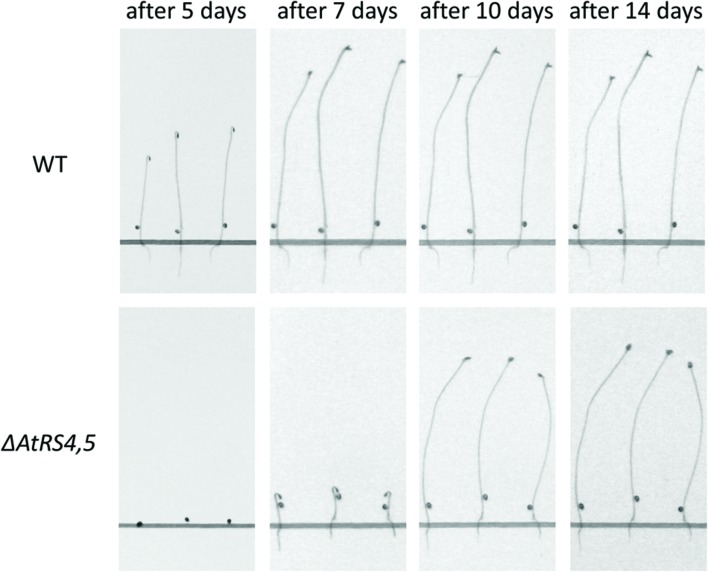
**Delayed *ΔAtRS4,5* seed germination phenotype in the dark.** Three representative seedlings are shown 5, 7, 10, and 14 days after sowing. Seedlings were grown on 0.5× MS plates in the dark.

**FIGURE 11:**
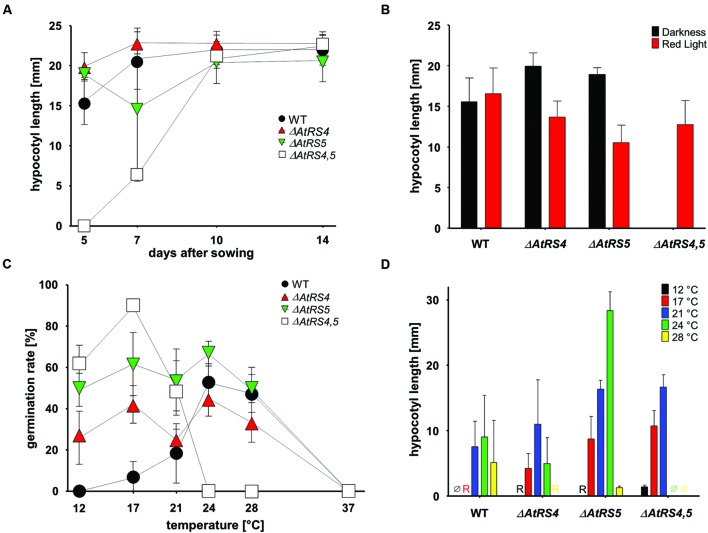
**Hypocotyl length and germination kinetics of *WT, ΔAtRS4*, *ΔAtRS5*, and *ΔAtRS4,5* plants. (A)** Hypocotyl length of germinated seeds in the darkness within 14 days. Values are averages of 20 to 30 performed measurements (±SD). **(B)** Hypocotyl length of germinated seeds treated either with red light for 5 days. Values are averages of 20 to 30 performed measurements (±SD). **(C)** Temperature dependent germination of seeds in darkness. Values are averages of three independently performed measurements (*n* = 20 to 30 seeds ±SD). **(D)** Hypocotyl length of germinated seeds in the darkness 5 days after sowing at different temperatures. Values are averages of 20 to 30 performed measurements (±SD). Ø indicates no visible protrusion and R indicates visible protrusion of the radicle under the binocular microscope. Values are averages of three independently performed measurements (±SD).

To test whether RFOs play a special role during early seed development when seeds are totally dependent on their accumulated resources, we put WT, *ΔAtRS4, ΔAtRS5*, and *ΔAtRS4,5* seeds on 0.8% water agarose, 0.5% water agar, on 0.3% water phytagel, or on 0.5× MS plates to let them germinate in darkness at approximately at 23°C for 7 days. Even after 14 days all seed types did not germinate on 0.8% water agarose, on 0.5% water agar or on 0.3% water phytagel plates, suggesting that nitrate as dormancy breaking signal is needed (Alboresi et al., 2005; Bethke et al., 2006). WT seeds germinated on 0.5× MS plates after 2 days. In contrast, *ΔAtRS4,5* seeds did not germinate on 0.5× MS plates in the dark until day 6 (**Figure 10**). Varying (0.3x and 0.8x) MS concentrations did not change the germination pattern. Continuing the experiment until the 14^th^ day after sowing, WT, *ΔAtRS4*, *ΔAtRS5*, and *ΔAtRS4,5* hypocotyls reached all the same length of about 22 mm (**Figures 11A,B**) suggesting that the RFO carbohydrate pool is not determining the maximal length of etiolated seedlings.

We carved out that seeds in the dark differ from WT seeds in a temperature dependent manner (**Figure 11C**). Within 5 days *ΔAtRS4,5* seeds were not able to germinate at temperatures over 24°C in the dark. *ΔAtRS4,5* seeds not only still germinated at lower temperatures compared to WT seeds at 12°C, but reached a maximum of germination rate at 17°C in the dark. The WT germination rate optimum was reached between 24 and 28°C. At 21°C, which was the only comparable temperature due to germination patterns, *ΔAtRS4,5* hypocotyl length almost doubled WT hypocotyl length (**Figure 11D**).

### Rescue of Delayed *ΔAtRS4,5* Seed Germination Phenotype in the Dark

We tested whether the addition of phytohormones (gibberellic acid, abscisic acid, and auxin) (Koornneef et al., 1982, 1984; Debeaujon and Koornneef, 2000), or nitrate (Alboresi et al., 2005) rescues the delayed germination phenotype in *ΔAtRS4,5* but none of these treatments lead to early germination of *ΔAtRS4,5* seeds in the dark. We further rationalized that most likely the sugars, being different in *ΔAtRS4,5* compared to WT, might be responsible for the delayed germination in the dark. The addition of Gal (1 g L^-1^) but not Raf, Sta, Gol, Suc, Glc, or fructose (Frc) (Garciarrubio et al., 1997) indeed partially rescued the delay in germination of *ΔAtRS4,5* seeds (**Table 1**).

**Table 1:** Partial rescue of delayed *ΔAtRS4,5* seed germination phenotype.

Sugar [1 g L^-1^]	Germination Rate [%]
Gal	23.1 ± 10.9
Raf	ND
Sta	ND
Gol	ND
Suc	ND
Glc	ND
Frc	ND

*ND, Not detectable.*

## Discussion

In this study, we cloned *AtRS5* cDNA from *A. thaliana* and heterologously expressed and purified the encoded functional protein from *E. coli*. In different enzyme assays we demonstrate that AtRS5 is a functional high affinity RafS (**Figures 3C,D**) as well as a Raf (**Figure 3E**) and Gol specific galactosylhydrolase (**Figure 3F**). The enzyme catalyzes the synthesis of Raf from Suc and Gol (Supplementary Figure S3). Beside the RafS enzyme activity, recombinant AtRS5 was able to hydrolyse Raf and Gol (**Figure 4**), but not Sta. On SDS gel, the molecular mass of the heterologously expressed recombinant AtRS5 was about 90 kDa, which corresponds fairly well with the calculated molecular weight of 86 kDa (**Figure 2**).

### Recombinant RafS Has Far Higher Catalytic Activity

Despite numerous experimental attempts to biochemically characterize RafSs (Supplementary Table S2) from various species (Lehle and Tanner, 1973; Castillo et al., 1990; Peterbauer et al., 2002; Li et al., 2007; Sui et al., 2012; Egert et al., 2013), in this study, it is the first time to our knowledge to purify a recombinant RafS from *A. thaliana* out of *E. coli* and characterize it biochemically. Recombinant AtRS5 enzyme displayed a much higher affinity toward Suc when assayed with Gol (*K*_m_ 0.35 mM), compared with K_m_ values of partially *PsRafS* (*K*_m_ between 7.3 and 22.6 mM; Peterbauer et al., 2002) or *VfRafS* (*K*_m_ 1 mM; Lehle and Tanner, 1973). Those high deviations within different K_m_ values are likely due to the fact, that all reported RafS kinetics so far were performed with purified enzyme from seeds or crude extracts from transformed cell lysate. We consider a possible posttranslational modification of RafS enzymes in previous reports, causing a strong reduction of substrate affinity, as rather unlikely. This might, however, to be analyzsed in future experiments. The affinity toward Gol was also much higher when assayed with Suc (*K*_m_ 0.55 mM), compared with estimated *K*_m_ values of *PsRafS* (*K*_m_ between 7.9 and 22.9 mM (Peterbauer et al., 2002)) and *VfRafS* (*K*_m_ 7 mM; Lehle and Tanner, 1973). Recombinant AtRS5 specific RafS activity [*V*_max_ (Suc) 2,050 pkat mg^-1^ protein and *V*_max_ (Gol) 2,554 pkat mg^-1^ protein] was comparable with specific RafS activity of *OsRafS* (V_max_ (Suc) 25.6 nkat mg^-1^ protein (Li et al., 2007)], *PsRafS* [*V*_max_ (Suc) between 0.8 to 199.2 pkat mg^-1^ protein (Peterbauer et al., 2002)], *GmRafS* [*V*_max_ (Suc) 0.7 nkat mg^-1^ protein (Castillo et al., 1990)], and *VfRafS* [*V*_max_ (Suc) 8.2 nkat mg^-1^ protein (Lehle and Tanner, 1973)]. This suggests that RafS have a high activity under physiological conditions and that the previous protein preparations had lost much of their substrate binding properties. We recently found the same phenomenon for StaS from *Arabidopsis*, where the K_m_ were about 70-fold lower for the recombinant enzyme compared to previous measurements in crude or partially purified enzyme extracts (Gangl et al., 2015). By heterologously expressing the *AtRS5* cDNA in *E. coli* Egert et al. (2013) demonstrated, that heterologously expressed crude extracts synthesize Raf, but no α-galactosidase activity on Raf. Contrary to these results, recombinant AtRS5 expressed in *E. coli* showed galactosylhydrolase activity on Raf and Gol. Raf specific [*V*_max_ (Gal) 2,093 pkat mg^-1^ protein] galactosylhydrolase activity of AtRS5 was comparable with high specific RafS activity. AtRS2 kinetics displayed comparable galactosylhydrolase activity [*V*_max_ 1.80 nkat mg^-1^ protein (Peters et al., 2010)] with AtRS5 kinetics, whereas substrate affinity of AtRS5 was much higher toward Raf [*K*_m_ (Gal) 2,712 μM], compared with *K*_m_ value of AtRS2 (*K*_m_ 105 mM), but still comparable with *OsRaf* [*K*_m_ 5.7 mM (Li et al., 2007)].

### Two AtRS Enzymes Contribute to Seed Storage RFOs

Little is known about the contribution to the RFO physiology in *A. thaliana* of the six annotated *AtRS* genes in the *Arabidopsis* genome (Peters et al., 2010; Egert et al., 2013). During *A. thaliana* seed development substantial quantities of Raf as well as Sta accumulate (Ooms et al., 1993; Bentsink et al., 2000; Nishizawa-Yokoi et al., 2008). A phylogenetic tree separated *AtRS4* and *AtRS5* in the group of RFO synthase. *ΔAtRS4* seeds showed an increased Raf concentration and a total loss of Sta accumulation (Gangl et al., 2015). *ΔAtRS5* seeds showed a decreased concentration of Raf of 0.5-fold and Sta concentrations were about equal in WT seeds (**Figure 7A**) (Egert et al., 2013). As already argued in Gangl et al. (2015), we hypothesized that seeds from *ΔAtRS4*,5 plants should be devoid of Raf and Sta. To confirm this hypothesis of *AtRS4* being the second seed-specific RafS, we crossed a *ΔAtRS4* and a *ΔAtRS5* plant and created a double-mutant *ΔAtRS4,5* plant. *ΔAtRS4,5* seeds showed a total loss of detectable Raf and Sta on HPLC. These results confirm our hypothesis that in *A. thaliana* seeds Sta accumulation is mediated by a single gene *AtRS4* (Gangl et al., 2015) and that Raf accumulation in seeds is mediated by both, *AtRS5* together with *AtRS4*. Interestingly we found elevated levels of Gol in unstressed leaves. RafS as well as StaS are enzymes which on one hand synthesize RFOs but on the other hand also hydrolyse RFOs and Gol. This is supported by our observation that upon stress relieve the RFO content lowers. A knockout of both enzymes might therefore interrupt the constant cycling of low RFO synthesis and degradation even in unstressed leaves with the consequence of increased Gol.

### AtRS4 and AtRS5 Are Not the Only RafS in *Arabidopsis*

Since reports provide conflicting data about the number of RafS contributing to Raf under stress, suggesting either the existence of a single (Zuther et al., 2004; Egert et al., 2013) or five (Nishizawa et al., 2008) abiotic stress-induced *AtRS* isoforms occurring in *Arabidopsis*, we analyzed the WSCs of WT, *ΔAtRS4*, *ΔAtRS5*, and *ΔAtRS4,5* plants as well as the expression of all five *AtRS* genes with sqPCR in WT plants under various abiotic stress conditions. After salt, cold (Klotke et al., 2004), and drought stress treatment, *AtRS5* was induced as expected (Egert et al., 2013). The expression profile also revealed that *AtRS6* was only induced by DS and is accompanied by Raf accumulation in drought stressed *ΔAtRS4,5* plants. These results suggest that *AtRS6* also contributes to RFOs under DS beside the major abiotic stress inducible isoform *AtRS5*. Different gene expression profiles of abiotic stress-induced GolS isoforms (*AtGS1-10*) - the hypothesized key regulatory enzymes of RFO biosynthesis upstream of RafS - were already shown by Taji et al. (2002). *AtGS1* and *AtGS2* are induced by drought and high-SS, whereas CS induces *AtGS3* (Supplementary Figure S7). The accumulation of RFOs in *Arabidopsis* leaves after different abiotic stress treatments appears to be the result of a coexpression network with *AtRS5* in its center. *AtRS5* is induced after each tested abiotic stress treatment (**Figure 8**) and therefore considered to be a key integrator of stress induced metabolic changes in RFO biosynthesis. Based on our results and the results shown by Taji et al. (2002), we hypothesize that *AtGS1*, *AtGS2*, and *AtMIPS2* together with *AtRS6* constitute a second, DS induced network beside the major abiotic stress inducible network with *AtRS5* (Supplementary Figure S7). Isolation of a *ΔAtRS6* plant as well as generation of a *ΔAtRS4,5,6* triple knockout plant would allow further insights into the metabolic reaction to different abiotic stresses.

### The Role of RFO in Germination

Germination experiments in the light with WT and *ΔAtRS4,5* seeds under SCC show similar germination kinetics with a slight but consistent earlier germination of the *ΔAtRS4,5* mutant. However, when the experiment was performed in the dark *ΔAtRS4,5* seeds did not germinate at all within 5 days in the dark whereas the WT seeds readily germinate to etiolated seedlings. However, after 14 days in the dark *ΔAtRS4,5* hypocotyls reached the same length as WT, *ΔAtRS4* and *ΔAtRS5*, suggesting that the RFO content of the seeds is not limiting the final length of etiolated seedling. Downie and Bewley (2000) hypothesized RFOs to play a special role during early seed germination, which our results support: The RFO content in seeds has a huge impact on the time point of the protrusion of the radicle and speeds up this early germination event. But RFOs are not required for a successful germination (Blöchl et al., 2007) neither do they influence the percentage of germination (Dierking and Bilyeu, 2009; Gangl et al., 2015) nor the hypocotyl length.

The germination kinetic of the *ΔAtRS4,5* seeds being earlier in the light but strongly delayed in the dark suggested the involvement of phytochrome in this process. Phytochrome dependent germination of seeds is mediated by a complex transcriptional network (Leivar and Monte, 2014) mediated by PIFs a subgroup of bHLH-transcription factors, which act as repressors of germination. Our finding that *AtPIF6* is much stronger expressed in *ΔAtRS4,5* than in WT seeds can thus explain the observed delay in germination only found in the dark. Under light conditions, *AtPIF6* is rapidly degraded in the proteasome after phytochrome mediated phosphorylation.

Water-soluble carbohydrates, like Suc (Stewart et al., 2011) and Glc (Dekkers et al., 2004) cause a delay in germination, while Suc concurrent leads to an increase in seedling length. Stewart et al. (2011) showed that Suc promoted growth requires the function of PIFs. The delayed germination phenotype in the dark can be partially rescued by Gal but not by other sugars. This suggests a model to us in which RFOs are considered as a storage complex of endogenous Gal similar to inositol hexaphosphate (IP_6_), phytic acid, is storage for phosphate. Starting with imbibition, α-galactosidase (like AtRS2) mobilizes Gal from the RFOs and Gal acts via *AtPIF6* transcription factor as an integrator and signal multiplier of germination-promoting events. If no RFO storage is present like in *ΔAtRS4,5* seeds, germination is delayed in the dark but not delayed in light, because phytochromes are active and continue to phosphorylate PIFs resulting in their degradation.

*AtPIF1* (Oh et al., 2004) and *AtPIF6* (Penfield et al., 2010) together with *AtSPT* (Penfield et al., 2005) (SPATULA, non-phy-interacting bHLH factor) affect seed germination. *AtPIF1* overexpression lines as well as *ΔAtPhyB* (Shinomura et al., 1994) did not germinate well in the dark, whereas the loss of *AtPIF6* increases dormancy in light and *AtSPT* suppresses germination in the absence of a cold treatment, regardless of light. A contribution of Raf accumulation to abiotic stress tolerance, especially freezing (Zuther et al., 2004) and drought tolerance (Taji et al., 2002), is often mentioned in literature, but highly to question (Zuther et al., 2004). Temperatures above 27°C lead to a complete elimination of light-induced *ΔAtPIF4* hypocotyl extension (Koini et al., 2009; Stavang et al., 2009). An accompanied fast increase in *AtPIF4* transcript suggest a temperature-sensing signal pathway, independent of the *AtPhy* pathway (Leivar and Quail, 2011). Johansson et al. (2014) showed that the classical light response of hypocotyl elongation is strictly temperature dependent. Our results showed a temperature dependent diametrically opposed germination rate of *ΔAtRS4,5* and WT seeds in the dark (**Figure 11C**). Based on their work and our results we demonstrated that a shift in temperature induces a reversal of response from inhibition to promotion of germination. And we hypothesize that seed RFOs interacting with PIF-transcription factors integrate exogenous and endogenous factors, respectively, control temperature and light dependent germination.

## Author Contributions

Conceived and designed the experiments: RG and RT. Performed the experiments: RG and RT. Analyzed the data: RG and RT. Contributed reagents/materials/analysis tools: RG and RT. Wrote the paper: RG and RT.

## Conflict of Interest Statement

The authors declare that the research was conducted in the absence of any commercial or financial relationships that could be construed as a potential conflict of interest.
