# Supplementary material for: Raffinose Family Oligosaccharides Act As Galactose Stores in Seeds and Are Required for Rapid Germination of Arabidopsis in the Dark
Source: Front Plant Sci. 2016 Jul 26;7:1115. doi: 10.3389/fpls.2016.01115 (PMC4960254; doi:10.3389/fpls.2016.01115)
Supplement: Supplementary file 1 [file Data_Sheet_1.DOCX]

Supplementary Material

**Raffinose family oligosaccharides act as galactose stores in seeds and are required for rapid germination of Arabidopsis in the dark**

Roman Gangl, Raimund Tenhaken^*)^

Department of Cell Biology, Plant Physiology, University of Salzburg, Salzburg, Austria

**^*)^ Correspondence:**

Raimund Tenhaken

Division of Plant Physiology

Department of Cell Biology

University of Salzburg

Hellbrunnerstrasse 34,

5020 Salzburg, Austria

raimund.tenhaken@sbg.ac.at

# Supplementary Figures and Tables

## Supplementary Figures


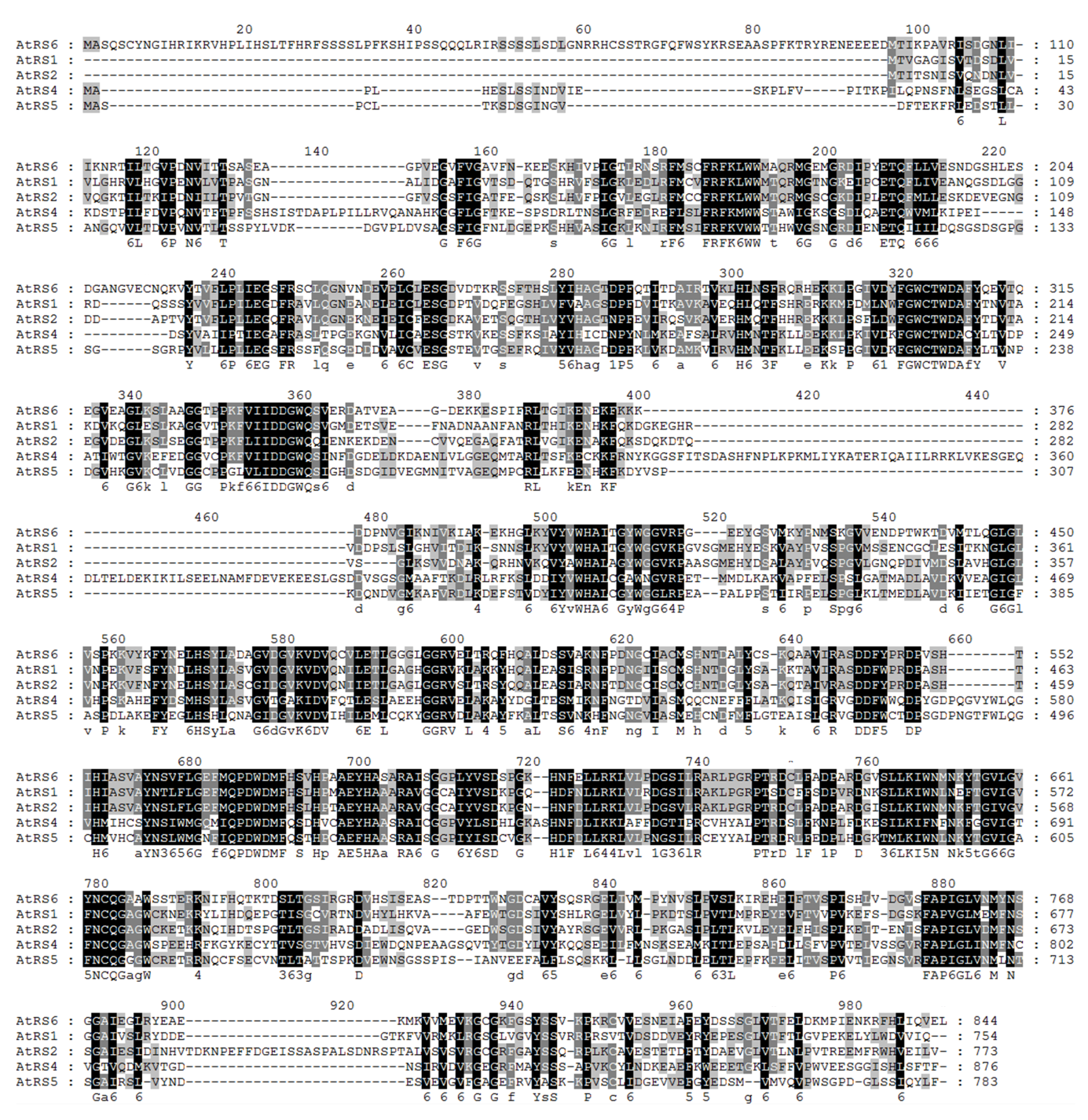


**Supplementary Figure 1** Sequence alignment of *AtRS* isoforms. A sequence alignment was performed with Clustal Omega (Sievers et al., 2011) of all 5 *AtRS* amino acid sequences. AtRS1, gi|15222768; AtRS2, gi|15230330; AtRS4, gi|332656706; AtRS5, gi|332195171; and AtRS6, gi|334187792.


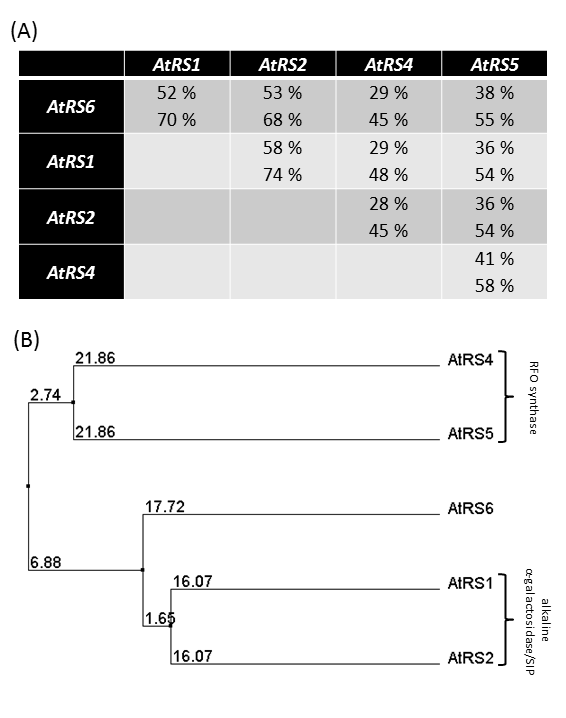


**Supplementary Figure 2** Sequence and phylogenetic analysis of *AtRS* genes in Arabidopsis. (A) Identity and similarity were calculated with GeneDoc (Nicholas et al., 1999) and (B) phylogenetic distance tree (Average Distance Using % Identity) was calculated with JalView (Waterhouse et al., 2009). AtRS1, gi|15222768; AtRS2, gi|15230330; AtRS4, gi|332656706; AtRS5, gi|332195171; and AtRS6, gi|334187792.


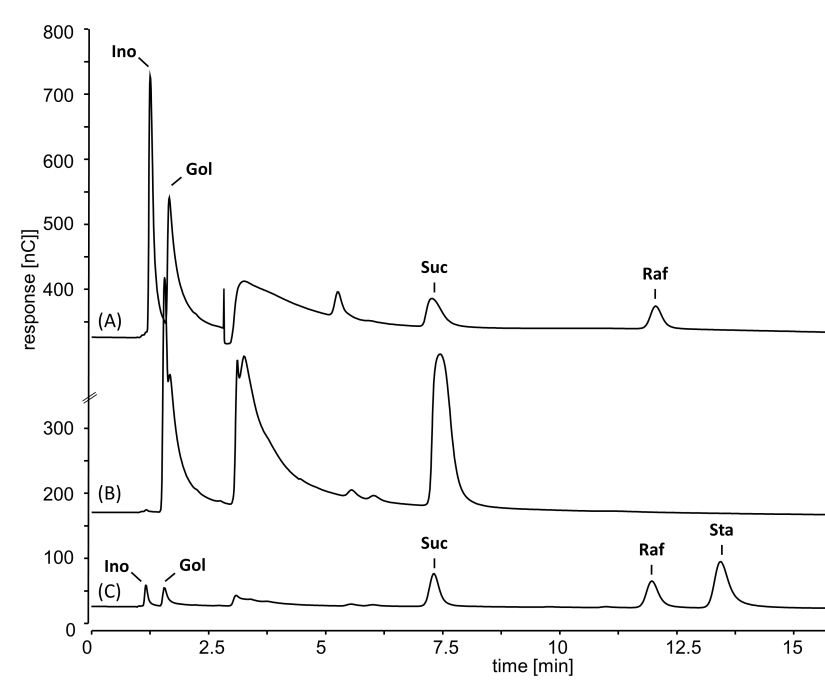


**Supplementary Figure 3** HPAEC-PAD enzyme assay. RafS enzyme activity of recombinant AtRS5 was tested. HPAEC-PAD chromatogram (A) indicates Raf product formation during enzyme reaction. HPAEC-PAD chromatogram (B) shows control without recombinant AtRS5. HPAEC-PAD chromatogram (C) shows 100 µM Ino, Gol, Suc, Raf and Sta as reference compounds.


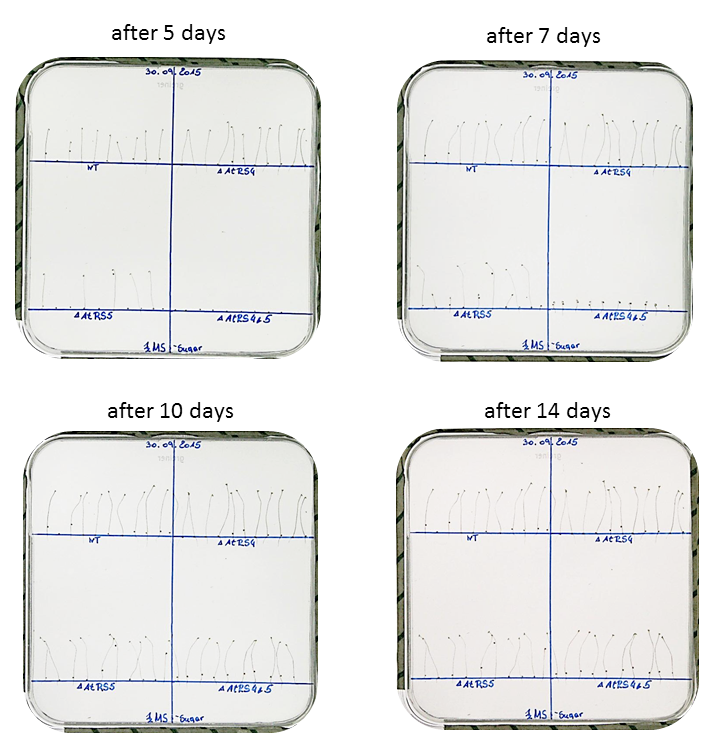


**Supplementary Figure 4** Germination of WT, *ΔAtRS4*, *ΔAtRS5* and *ΔAtRS4*,5 seeds on 0.5 x MS agar plates in darkness. Plates were incubated for 5, 7, 10 and 14 days in darkness.


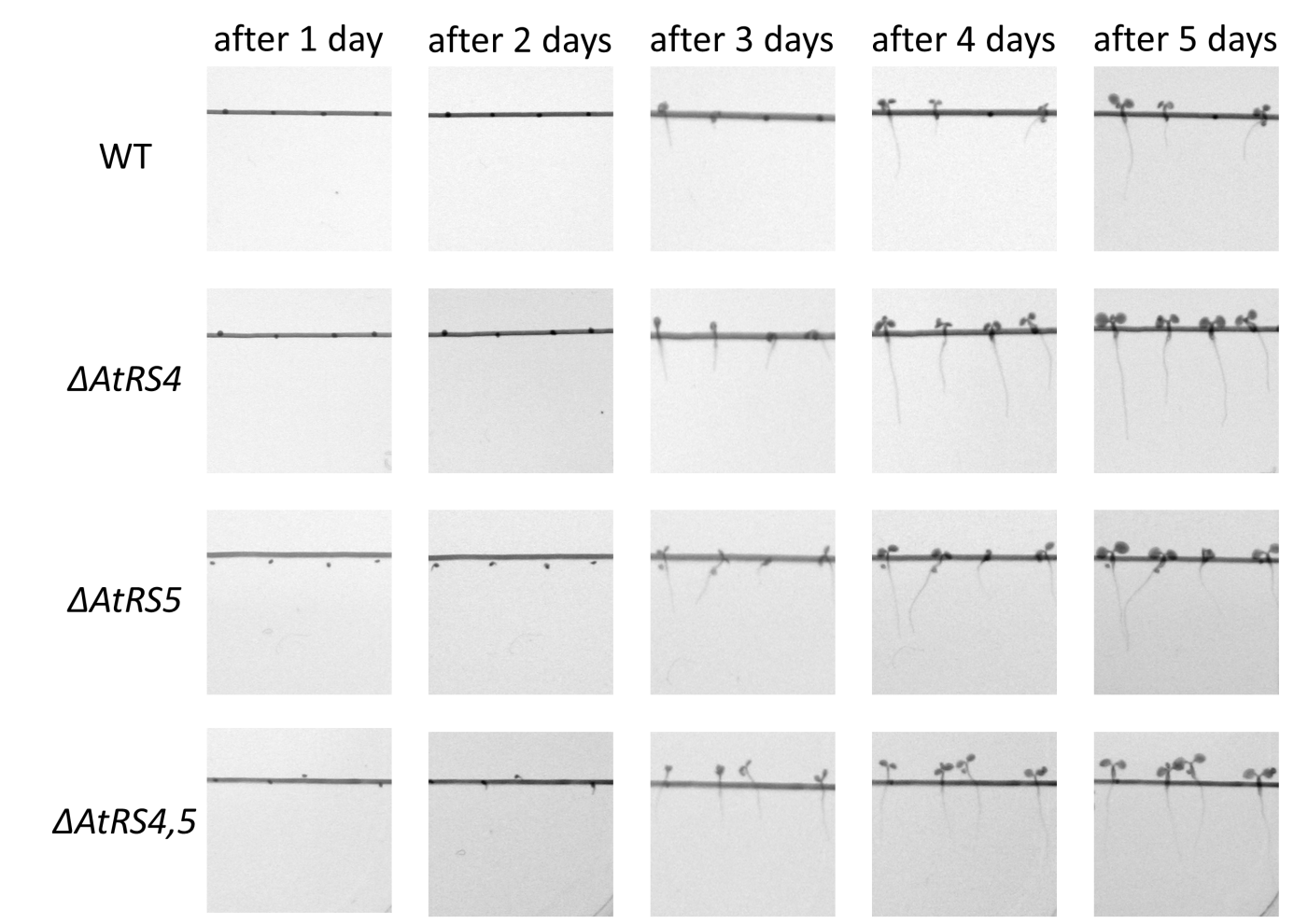


**Supplementary Figure 5** Germination of WT, *ΔAtRS4*, *ΔAtRS5* and *ΔAtRS4*,5 seeds on 0.5 x MS agar plates under SCC. Plates were incubated for 1, 2, 3, 4 and 5 days under SCC.


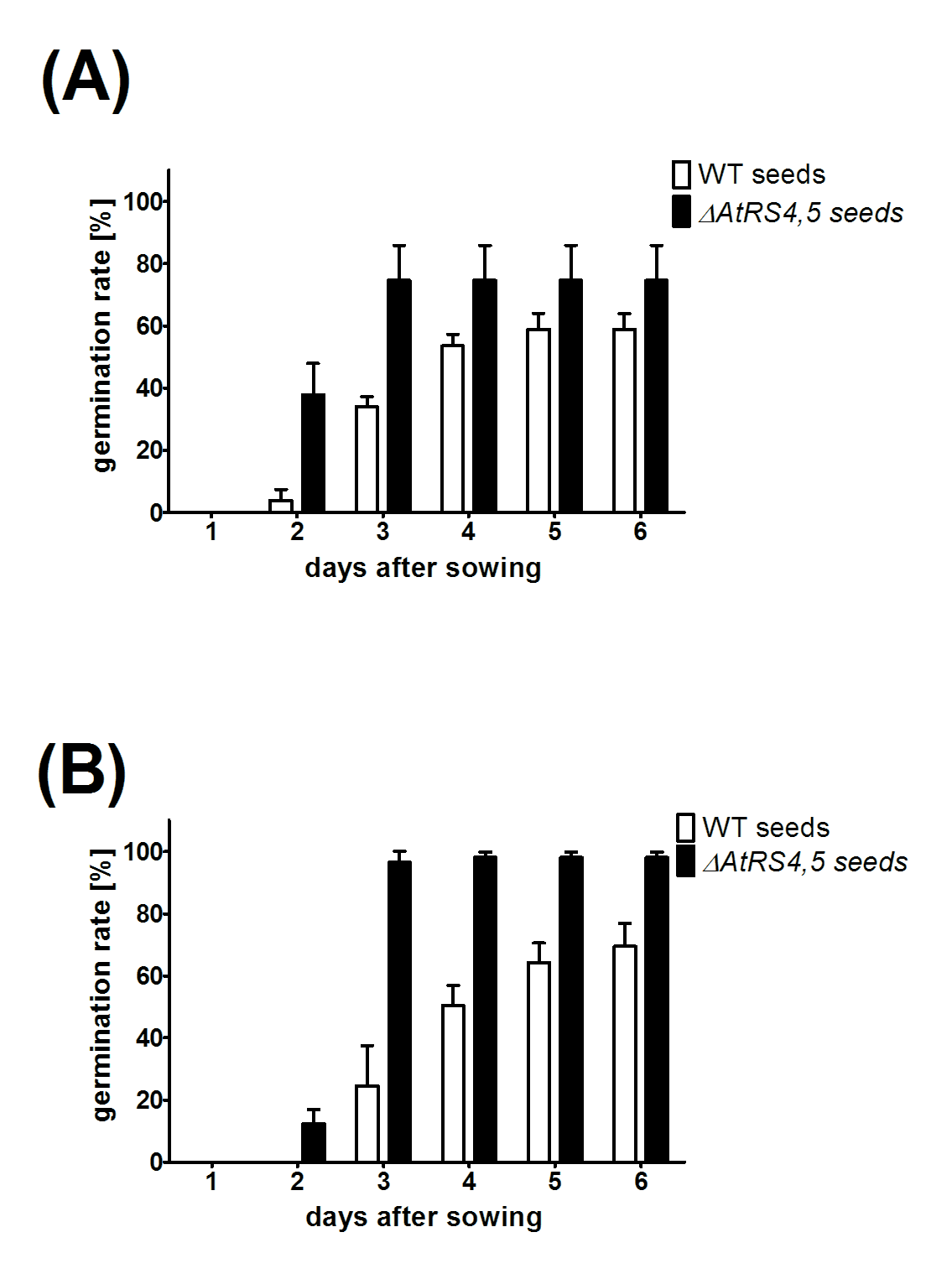


**Supplementary Figure 6** Kinetic of WT and *ΔAtRS4,5* seed germination. WT and *ΔAtRS4,5* mutant seeds for germination experiment were sowed either on (A) 0.5 % agarose or (B) 0.5 % plant agar and grew under control growth conditions. Germination was defined as the time between sowing and protrusion of the radicle. Results indicate an impact of the plate media probably due to its moisture levels on the maximum percentage of germination, but not on germination kinetics itself. Values are averages of three independently performed experiments (+/- SD).

**
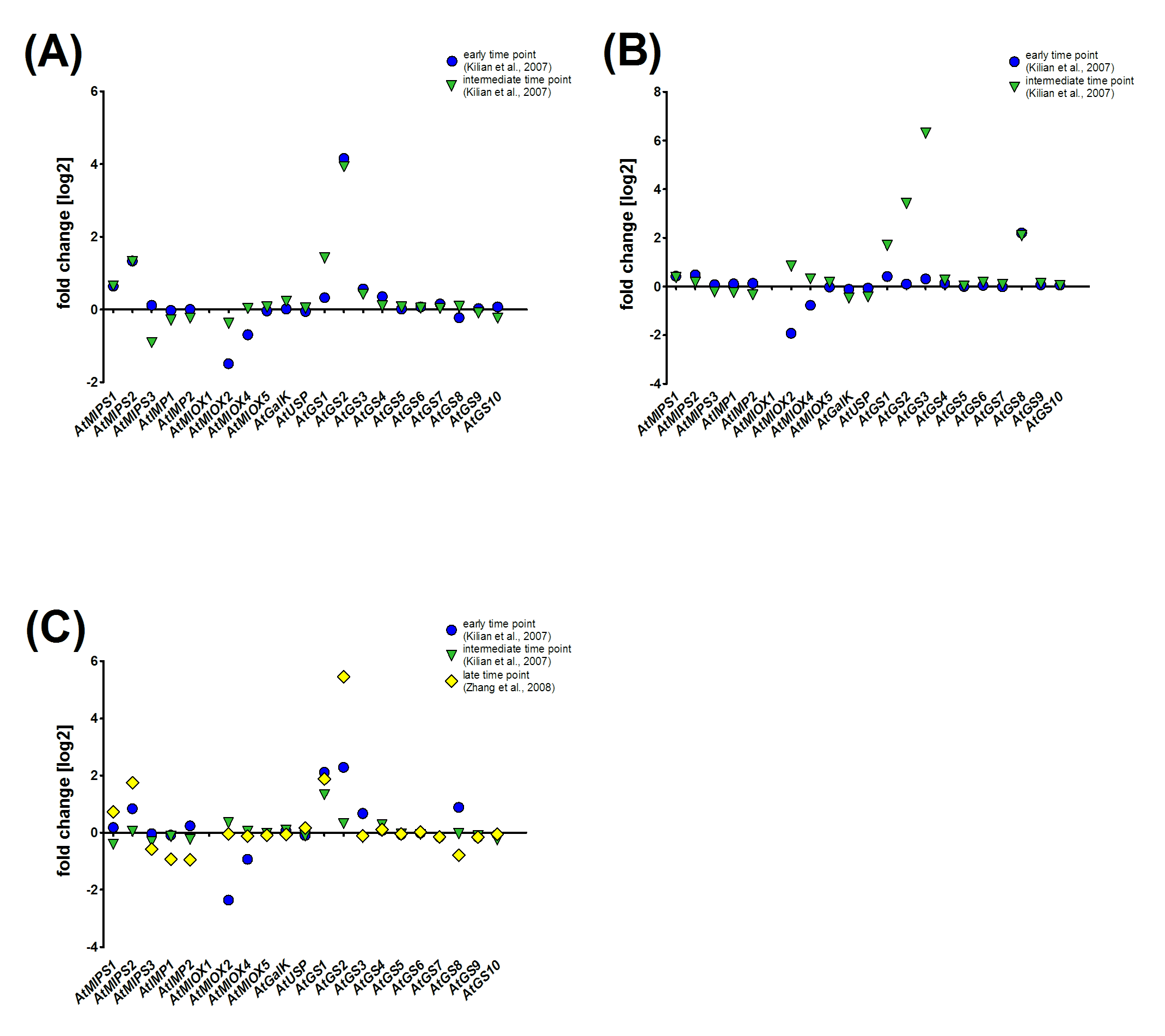
**

**Supplementary Figure 7** **G**ene expression analysis with Genevestigator. The log2 fold change values are depicted after (A) salt, (B) cold and (C) drought stress treatment. Gene expression data points are derived from microarray data from AtGenExpress (Schmid et al., 2005;Kilian et al., 2007) (early (up to 1 h) and intermediate (up to 24 h) time point from salt stress time course; early and intermediate time point from cold stress time course; early and intermediate time point from drought stress time course) and from Zhang et al. (2008) (late (up to 11 days) time points from drought stress) as verification. *AtMIPS1* (AT4G39800), *AtMIPS2* (AT2G22240), *AtMIPS3* (AT5G10170), *AtIMP1* (AT1G31190), *AtIMP2* (AT4G39120), *AtMIOX1* (AT1G14520), *AtMIOX2* (AT2G19800), *AtMIOX4* (AT4G26260), *AtMIOX5* (AT5G56640), *AtGalK* (At3g06580), *AtUSP* (At5g52560), *AtGS1* (At2g47180), *AtGS2* (At1g56600), *AtGS3* (At1g09350), *AtGS4* (At1g60470), *AtGS5* (At5g23790), *AtGS6* (At4g26250), *AtGS7* (At1g60450), *AtGS8* (At3g28340), *AtGS9* (At3g06260), *AtGS10* (At5g30500).

## Supplementary Tables

**Supplementary Table 1** Primer sequences.

| Primer | Sequence |
| --- | --- |
| wtAtRS4_fwd | 5′-AAGGAGGGTTTCTCGGATTC-3’ |
| wtAtRS4_rev | 5′-TCTCAGCGTCTTTGTCTAGC-3’ |
| SALK left border | 5′-ACTCAACCCTATCTCGGGCTATTC-3’ |
| wtAtRS5_fwd | 5’-AATGGCTTCGCCGTGTTTG-3’ |
| wtAtRS5_rev | 5’-AAACTCCGACCCGGTTACTTC-3’ |
| GABI-Kat left border | 5’-TTTCTCCATATTGACCATCATACTCATTG-3’ |
| AtRS5_fwd | 5`-AAGGTACCATGGCTTCGCCGTGT-3’ |
| AtRS5_rev | 5’-AACCCGGGCTAAAACAAATACTGAATAGAAGACAA-3’ |
| qAtEF1α_fwd | 5’-GACCAACTCTTCTTGAGGCTCTTGAC-3’ |
| qAtEF1α_rev | 5’-GGCACCGTTCCAATACCACCAATC-3’ |
| sqAtRS1_fwd | 5’-TGTCATTACCAAAGCTGTCAAGGC-3’ |
| sqAtRS1_rev | 5’-CAAGTGCACCAACCAAACCAATTC-3’ |
| sqAtRS2_fwd | 5’-TAAAATGCGCCGTTGAAAGC-3’ |
| sqAtRS2_rev | 5’-TCGTCACCGGCAGATTCAAAG-3’ |
| sqAtRS4_fwd | 5’-GCTGGGATTGGTCTTGTTCATCC-3’ |
| sqAtRS4_rev | 5’-TTCTGCTAGTGACTCCAAGGTTTG-3’ |
| sqAtRS5_fwd | 5’-GTCTGGTCCAGATGGTTTGTCTTC-3’ |
| sqAtRS5_rev | 5’-CAACGAACGGTACGGAGTTAAG-3’ |
| sqAtRS6_fwd | 5’-GTCTCACTCAAAATCCGTGAGCAC-3’ |
| sqAtRS6_rev | 5’-TCGATAGCTCCTCCCGAATTG-3’ |
| qAt1g17830_fwd | 5’-TCCGGGACTTTCGACTCTAAGGAG-3’ |
| qAt1g17830_rev | 5’-TGGATACCAAGCAATGGCCAACC-3’ |
| qAt1g75450_fwd | 5’-ACTCCGAAATCGTTGATCAGGAAG-3’ |
| qAt1g75450_rev | 5’-GGAGCTTCAATTCGGCCTTGTG-3’ |
| qAt2g20180_fwd | 5’-ACTTATACCTCGCTGCAACAAGTC-3’ |
| qAt2g20180_rev | 5’-CATGCCCGGATACATCATTGGC-3’ |
| qAt3g16150_fwd | 5’-ACGGACGACAACGTAGGAATGC-3’ |
| qAt3g16150_rev | 5’-GGCGGAATCCGGTAATCAAACAAG-3’ |
| qAt3g62090_fwd | 5’-TCACCTGAGAGAGACGACAACG-3’ |
| qAt3g62090_rev | 5’-ACCCATCGTCATCATCTGAACTTG-3’ |
| qAt5g02200_fwd | 5’-GACTCCGAATTTGCGGTCGAAG-3’ |
| qAt5g02200_rev | 5’-TGGAATCTTGGTTGCTGACACTCC-3’ |

**Supplementary Table 2** Data of biochemical characterizations of RafSs.

| Plant species | Enzyme Source | Reaction | V_max_  [kat mg^-1^ protein] | pH opt. | T  [°C] | K_m_ Suc  [µM] | K_m_ Gol  [µM] | Reference |
| --- | --- | --- | --- | --- | --- | --- | --- | --- |
| *Arabidopsis thaliana* | purified recombinant enzyme  expressed in *E. coli* | Raf Synthesis  Raf Hydrolysis  Gol Hydrolysis | 2.0 10^-9^ to 2.5 10^-9^  2.0 10^-9^ | 6 | 20 to 25 | 348 | 548  2,700 | This paper |
| *Arabidopsis thaliana* | heterologously expressed  enzyme in *E. coli* crude extract | Raf Synthesis | n.d. | n.d. | n.d. | n.d. | n.d. | Egert et al. (2013) |
| *Cucumis sativus* | Agrobacterium-transformed  tobacco crude extract | Raf Synthesis | n.d. | n.d. | n.d. | n.d. | n.d. | Sui et al. (2012) |
| *Oryza sativa* | purified recombinant enzyme  expressed in *E. coli* | Raf Synthesis  Raf Hydrolysis  Gol Hydrolysis  *p*NP-α-Gal Hydrolysis | 25.6 10^-9^  5.7 10^-9^  8.5 10^-9^  6.8 10^-9^ | 7 | 45 | n.d. | n.d. | Li et al. (2007) |
| *Pisum sativum* | Baculovirus-infected insect  cell lysate | Raf Synthesis  Raf Synthesis^*)^ | 2.0 10^-12^  0.8 10^-12^ | 7 | n.d. | 22,600 | 7,900 | Peterbauer et al. (2002) |
|  | partially purified enzyme | Raf Synthesis  Gol Hydrolysis  Raf Synthesis^*)^ | 199.2 10^-12^  27.3 10^-12^  75.4 10^-12^ | 7 | n.d. | 7,300 | 22,900  1,000 |  |
| *Glycine max* | purified enzyme | Raf Synthesis^*)^ | 0.7 10^-9^ | 6.5 | n.d. | n.d. | n.d. | Castillo et al. (1990) |
| *Phaseolus vulgaris* | partially purified enzyme | Raf Synthesis | n.d. | n.d. | n.d. | n.d. | n.d. |  |
| *Vicia faba* | purified enzyme | Raf Synthesis^*)^  *p*NP-α-Gal Hydrolysis^*)^ | 8.2 10^-9^  0.2 10^-9^ | 7 | 42 | 1,000 | 7,000 | Lehle and Tanner (1973) |

^*)^ specific activity

**References**

Castillo, E.M., De Lumen, B.O., Reyes, P.S., and De Lumen, H.Z. (1990). Raffinose synthase and galactinol synthase in developing seeds and leaves of legumes. *Journal of Agricultural and Food Chemistry* 38**,** 351-355.

Egert, A., Keller, F., and Peters, S. (2013). Abiotic stress-induced accumulation of raffinose in Arabidopsis leaves is mediated by a single raffinose synthase (RS5, At5g40390). *BMC Plant Biol* 13**,** 218.

Lehle, L., and Tanner, W. (1973). The Function of myo‐Inositol in the Biosynthesis of Raffinose. *European Journal of Biochemistry* 38**,** 103-110.

Li, S., Li, T., Kim, W.-D., Kitaoka, M., Yoshida, S., Nakajima, M., and Kobayashi, H. (2007). Characterization of raffinose synthase from rice (Oryza sativa L. var. Nipponbare). *Biotechnology Letters* 29**,** 635-640.

Nicholas, K., Nicholas Jr, H., and Deerfield, D. (1999). II (1997) GeneDoc: analysis and visualization of genetic variation. *Embnew. News* 4**,** 14.

Peterbauer, T., Mach, L., Mucha, J., and Richter, A. (2002). Functional expression of a cDNA encoding pea (Pisum sativum L.) raffinose synthase, partial purification of the enzyme from maturing seeds, and steady-state kinetic analysis of raffinose synthesis. *Planta* 215**,** 839-846.

Sievers, F., Wilm, A., Dineen, D., Gibson, T.J., Karplus, K., Li, W., Lopez, R., Mcwilliam, H., Remmert, M., and Söding, J. (2011). Fast, scalable generation of high‐quality protein multiple sequence alignments using Clustal Omega. *Molecular Systems Biology* 7**,** 539.

Sui, X.-L., Meng, F.-Z., Wang, H.-Y., Wei, Y.-X., Li, R.-F., Wang, Z.-Y., Hu, L.-P., Wang, S.-H., and Zhang, Z.-X. (2012). Molecular cloning, characteristics and low temperature response of raffinose synthase gene in Cucumis sativus L. *Journal of Plant Physiology* 169**,** 1883-1891.

Waterhouse, A.M., Procter, J.B., Martin, D.M., Clamp, M., and Barton, G.J. (2009). Jalview Version 2—a multiple sequence alignment editor and analysis workbench. *Bioinformatics* 25**,** 1189-1191.
